# Gestational Diabetes Mellitus among Pregnant Women Delivering in a Tertiary Care Hospital: A Descriptive Cross-sectional Study

**DOI:** 10.31729/jnma.7304

**Published:** 2022-03-31

**Authors:** Sumi Singh, Manisha Yadav

**Affiliations:** 1 Nepal Medical College and Teaching Hospital, Jorpati, Kathmandu, Nepal

**Keywords:** *diabetes mellitus*, *gestational diabetes*, *pregnancy in diabetes*

## Abstract

**Introduction:**

Gestational diabetes mellitus is increasing globally leading to significant maternal and foetal morbidity. This study aimed to find out the prevalence of gestational diabetes mellitus among pregnant women delivering in a tertiary care hospital.

**Methods:**

A descriptive cross-sectional study on a total of 3034 pregnant women was conducted in a tertiary care hospital from 14^th^ April 2017 to 13^th^ April 2018 with ethical approval from Research and Institutional Review Committee (Reference number: 061-077/078) of the hospital. Pregnant women who met the eligibility criteria were included in the study. Convenience sampling was done. Data were analysed using the Statistical Package for the Social Sciences version 24.0 and Microsoft Excel. Point estimate at 99% Confidence Interval was calculated along with frequency and percentage for binary data.

**Results:**

Among 3034 patients who delivered in the tertiary centre, the prevalence of gestational diabetes mellitus was found to be 104 (3.42%) (2.57-4.26 at 99% Confidence Interval). The majority of women were of maternal age >30 years in 69 (66.34%). Out of which 48 (46.15%) women had family history of diabetes mellitus. Thirty-eight (36.53%) patients required a caesarean section. The most common obstetric complication was polyhydramnios in 35 (35.57%).

**Conclusions:**

The prevalence of gestational diabetes was lower when compared to other studies done in similar settings. The majority of women were of higher maternal age, had family history of diabetes mellitus and were also obese.

## INTRODUCTION

Gestational diabetes mellitus (GDM) is defined as carbohydrate intolerance of varying degrees with hyperglycemia starting during pregnancy.^1^

Diabetes during pregnancy has been associated with several maternal and foetal problems especially in developing countries like Nepal, which can lead to significant morbidity and mortality.^2-4^ The effect and burden of the disease are still under study and information regarding the epidemiology are still lacking.

The goal of this study was to find the prevalence of gestational diabetes mellitus among all pregnant women who delivered during the study period in a tertiary care centre.

## METHODS

This was a descriptive cross-sectional study conducted in the Department of Obstetrics and Gynaecology of Nepal Medical College and Teaching Hospital for a duration of one year from 14^th^ April 2017 to 13^th^ April 2018. The Nepal Medical College Institutional Review Committee (Reference number: 061-077/078) provided ethical clearance for the study. All the women who delivered at gestational age >28 weeks during the study period were included. Pregnant women with previous known case of diabetes mellitus and multiple gestations were excluded from this study. It was hospital protocol to subject all women admitted for delivery in the centre to fasting and postprandial blood glucose level and all women who met the eligibility criteria were included in the study. A convenience sampling technique was used.

The sample size was calculated using the following formula:

n = (Z^2^ × p × q) / e^2^

  = (2.57^2^ × 0.066 × 0.934) / 0.02^2^

  = 1018

Where,

n = minimum required sample size
Z = 2.57 at 99% Confidence Interval (CI)
p = prevalence of gestational diabetes mellitus was taken as 6.6%^5^
q = 1-p
e = margin of error, 2%

Adding a non-response rate of 10%, the final sample size was 1131. Since, convenience sampling method was used, the sample size was doubled resulting in 2262. However, a total of 3034 who delivered in the tertiary centre were included in the study.

Our study participants were pregnant women delivered in Nepal Medical College and Teaching Hospital during the study period. Data was collected from the antenatal records which included baseline characteristics, clinical risk factors, antenatal complications, including intrapartum and postpartum maternal problems, as well as numerous newborn issues, were examined in labour and delivery records of individuals diagnosed with GDM.

Data was analysed using the Statistical Package for the Social Science (SPSS) version 24.0 and results were tabulated in Microsoft Excel spreadsheet. Point estimate at 99% Confidence interval was calculated. Descriptive analysis such as frequencies, means, and percentages was used to present the collected data.

## RESULTS

Among all the 3034 patients who delivered in the institution, the prevalence of gestational diabetes mellitus was 104 (3.42%) (2.57-4.26 at 99% Confidence Interval). This study showed that the mean maternal age of women with GDM was 29.0±4.9 years and the mean gestational age at diagnosis was 25.1±9.5 weeks of gestation. The mean body mass index (BMI) among women with GDM in the study was 24.3 ±4.4. Around 45 (43.26%) women were nulliparous. The most clinically significant risk factors observed during the study were age >30 years in about 69 (66.34%) of women, followed by family history of diabetes mellitus in 48 (46.15%) and obesity in around 41 (39.42%). Previous history of foetal macrosomia was present in 3 (2.88%) patients and previous history of stillbirth in 1 (0.96%) patient (Table 1).

**Table 1:** Baseline characteristics of pregnant women in this study (n = 104).

Characteristics	Mean±SD
Maternal age (years)	29.0±4.9
Gestational age at diagnosis (weeks)	25.1±9.5
BMI (kg/m^2^)	24.3±4.4
**Parity**	n (%)
0	45 (43.26)
1	37 (35.57)
2	22 (21.15)
**Risk for GDM**	**n (%)**
Family history of DM	48 (46.15)
Maternal age >30 years	69 (66.34)
Previous history of fetal macrosomia	3 (2.88)
Previous history of stillbirth	1 (0.96)
Obesity	41 (39.42)

The mean gestational age at delivery was 39.6±1.2 weeks and among which 17 (16.34%) were preterm delivery while 87 (83.65%) of them were term delivery. The majority of the cases had normal vaginal delivery 66 (63.46%) and about 38 (36.53%) had a caesarean section which was significantly higher. The mean birth weight calculated of the infant was 2890.3±438.0 grams (Table 2).

**Table 2:** Labor and delivery data (n= 104).

Characteristics	Mean±SD
**Gestation age at birth (weeks)**	39.6±1.2
**Birth weight (gm)**	2890.3±438.0
**Gestation age at birth (weeks)**	**n (%)**
Preterm	17 (16.34)
Term	87 (83.65)
**Mode of delivery**	**n (%)**
Normal vaginal delivery	66 (63.46)
Cesarean section	38 (36.53)

The most common obstetric complication was polyhydramnios in 37 (35.57%) and neonatal complication was respiratory distress syndrome in 17 (16.34%) cases followed by stillbirth in 2 (1.92%) cases (Table 3). Other complications like shoulder dystocia, jaundice, and hypoglycemia were not observed. Large for gestational age infants were found in 28 cases (26.92%) while small for gestational age infants was found in 1 (2.88%) case. Around 89 (85.57%) of the infants had an APGAR score of more than seven while only 15 (14.42%) of the infants had a low APGAR score below seven at five minutes of birth.

**Table 3:** Obstetric and neonatal outcomes (n = 104).

Outcomes	n (%)
**Stillbirth**	2 (1.92)
**Respiratory Distress Syndrome**	17 (16.34)
**Apgar score at 5 minutes**	
>7	89 (85.57)
<7	15 (14.42)
**Size**	
Appropriate for gestational age	73 (70.19)
Small for gestational age	3 (2.88)
Large for gestational age	28 (26.92)
Polyhydramnios	37 (35.57)

## DISCUSSION

Gestational diabetes is one of the most common pregnancy problems, affecting up to five percent of all expecting mothers.^6^ The most recent GDM research examines the link between maternal hyperglycemia and an increased risk of negative pregnancy outcomes, as well as if treating the condition can reduce perinatal morbidity.^7^ The rising prevalence of gestational diabetes, its consequences on individual mothers and infants, and its public health implications have become serious concerns.^8^ The total number of GDM cases in our study was 104 (3.42%) among 3034 patient who delivered in the institution according to WHO criteria. Recent study suggests the incidence of GDM to be 6.6% in Nepal.^5^ Higher maternal glucose concentrations were found to be associated with worse pregnancy outcomes in the Hyperglycemia and Adverse Pregnancy Outcome (HAPO) research. In the current study,^9^ women with GDM showed a great percentage of obstetric difficulties, including caesarean section and neonatal complications such as respiratory distress syndrome, stillbirth, and macrosomic infants. Some researchers found a strong link between maternal-perinatal problems like foetal macrosomia, caesarean delivery, newborn hypoglycemia, hypertensive syndromes, and fasting glucose concentrations above 126 gm/dl.^10^

The most common potential clinical risk factor for GDM observed in this study was maternal age of more than 30 years. Increased maternal age is an important risk factor for the development of GDM.^11^ Similar studies show that increased BMI in adolescence and pregnancy have a threefold increased risk of having GDM.^12,13^ Foetal hyperglycemia causes increased osmotic diuresis, which causes polyuria and then polyhydramnios. This is backed up by a strong link between polyhydramnios and high glycosylated haemoglobin levels.^14^ In GDM, the foetus is persistently affected by high glucose levels from the maternal circulation. This leads to an elevated level of foetal insulin as maternal insulin itself cannot cross the placenta. The anabolic effects of insulin can lead to macrosomia.^15^ A study done in Madrid found macrosomia to be more common in children of mothers who had developed gestational diabetes mellitus. Another study also reported that macrosomia was more common in children of women who had developed diabetes mellitus after gestational diabetes mellitus.^16^

Macrosomia is linked to a higher rate of caesarean section, shoulder dystocia, chorioamnionitis, severe perineal lacerations, and postpartum haemorrhage, among other obstetric problems.^17^ Women with GDM had a greater rate of stillbirths than women with normal glucose tolerance in urban Iranian population research,^18^ which is comparable to the current study. Despite maternal blood glucose control throughout pregnancy, the rate of Caesarean section in GDM patients is relatively high, according to much research.^19,20^ Similarly, our study found that those with GDM have a high rate of caesarean operations, which is consistent with a previous study that found that women with GDM have a higher rate of caesarean deliveries because their infants are typically large for gestational age and are at a higher risk of prematurity.^21^ Prematurity linked to pregnancies affected by glucose intolerance could explain why GDMs have a greater rate of stillbirths. Meanwhile, the caesarean section rate was found to be 36.53% in this study. Women with GDM are more prone to have stillborn or macrosomic newborns, which may necessitate moderate or significant surgical intervention such as caesarean section.

Obese women and women who have had a previous cesarean section are more likely to acquire gestational diabetes during pregnancy. The outcomes of this study highlight the importance of increasing GDM screening in pregnant women and implementing interventions to mitigate the condition's consequences on both the infant and the mother.^22^ Similarly, another study found that an increased baseline fasting glucose during pregnancy was substantially predictive of big infant birth.^19^ On the other hand, a study discovered that rising postprandial glucose levels were linked to foetal macrosomia.^23^ Nutritional therapy, exercise, blood glucose monitoring, and insulin therapy are all common ways to control diabetes. Treatment lowered the chance of foetal overgrowth, according to multicenter randomised research.^24^ Insulin is the therapy of choice for any kind of diabetes during pregnancy. The majority of today's insulin formulations are safe and aid in glycemic control during pregnancy. According to certain research, even little alterations in glucose tolerance can cause aberrant foetal growth, which can be avoided with basic blood glucose regulation.^25^ A study has shown that, an exercise intervention can reduce macrosomia by 58 percent.^26^ To minimise serious maternal-foetal problems, we should actively employ insulin and health education to increase mother compliance. GDM's negative outcomes are unavoidable, but they can be reduced with preventive interventions, according to this study, which revealed caesarean section 36.53%, preterm delivery 16.34%, polyhydramnios 35.57%, and macrosomia 26.92%. As a result, meticulous blood glucose monitoring, dietary control, and insulin therapy with a low-risk profile have been regarded as the gold standard in the treatment of GDM. To determine unfavourable pregnancy outcomes in GDM, more prospective trials are needed. Early detection and treatment of these pregnant women and their newborn infants could improve their prognosis and avoid associated morbidity and mortality.

This was a single centre based study with small sample size so the results might not be generalizable to all target populations. As this was a descriptive cross-sectional study, comparison between groups could not be done and association with risk factors could not be established. Neonates were not followed up to monitor the foetal growth outcome; hence further study is required for proper management and to reduce the adverse maternal and foetal outcome due to this condition.

## CONCLUSIONS

The prevalence of gestational diabetes was lower when compared to other studies done in similar settings. The majority of women were of higher maternal age, had family history of diabetes mellitus and were also obese. The higher number of women with GDM underwent caesarean section and have polyhydramnios, preterm delivery and macrosomia in the neonates. Early detection, proper medication, and lifestyle changes could help to mitigate the negative effects. To address these issues and give more information about this condition on risk factors and potential outcomes, higher levels of study design should be done.
